# Carbon dioxide and hydrogen adsorption study on surface-modified HKUST-1 with diamine/triamine

**DOI:** 10.1038/s41598-022-22273-2

**Published:** 2022-10-17

**Authors:** Tomas Zelenka, Klaudia Simanova, Robin Saini, Gabriela Zelenkova, Satya Pal Nehra, Anshu Sharma, Miroslav Almasi

**Affiliations:** 1 Department of Chemistry, Faculty of Science, University of Ostrava, 30. Dubna 22, 701 03 Ostrava, Czech Republic; 2 Department of Inorganic Chemistry, Faculty of Science, P.J. Safarik University, Moyzesova 11, 040 01 Kosice, Slovak Republic; 3 Department of Physics, School of Engineering & Technology, Central University of Haryana, Mahendergarh, 123031 India; 4 Center of Excellence for Energy and Environmental Studies, Deenbandhu Chhotu Ram University of Science and Technology, Murthal, 131039 India

**Keywords:** Inorganic chemistry, Physical chemistry, Chemistry

## Abstract

The present article intended to study the influence of post-synthetic modification with ethylenediamine (*en*, diamine) and diethylenetriamine (*deta*, triamine) within the coordinatively unsaturated sites (CUSs) of HKUST-1 on carbon dioxide and hydrogen storage. The as-sythesized adsorbent was solvent-exchanged and subsequently post-synthetically modified with di-/triamines as sources of amine-based sorption sites due to the increased CO_2_ storage capacity. It is known that carbon dioxide molecules have a high affinity for amine groups, and moreover, the volume of amine molecules itself reduces the free pore volume in HKUST-1, which is the driving force for increasing the hydrogen storage capacity. Different concentrations of amines were used for modification of HKUST-1, through which materials with different molar ratios of HKUST-1 to amine: 1:0.05; 1:0.1; 1:0.25; 1:0.5; 1:0.75; 1:1; 1:1.5 were synthesized. Adsorption measurements of carbon dioxide at 0 °C up to 1 bar have shown that the compounds can adsorb large amounts of carbon dioxide. In general, *deta*-modified samples showed higher adsorbed amounts of CO_2_ compared to *en*-modified materials, which can be explained by the higher number of amine groups within the *deta* molecule. With an increasing molar ratio of amines, there was a decrease in wt.% CO_2_. The maximum storage capacity of CO_2_ was 22.3 wt.% for HKUST-1: *en*/1:0.1 and 33.1 wt.% for HKUST-1: *deta*/1:0.05 at 0 °C and 1 bar. Hydrogen adsorption measurements showed the same trend as carbon dioxide, with the maximum H_2_ adsorbed amounts being 1.82 wt.% for HKUST-1: *en*/1:0.1 and 2.28 wt.% for HKUST-1: *deta*/1:0.05 at − 196 °C and 1 bar.

## Introduction

Carbon dioxide emissions caused by anthropogenic activity, in particular electricity generation, industrial production and transport, account for about 80% of greenhouse gas emissions. For this reason, it is necessary to reflect and re-evaluate the currently used technologies and develop CO_2_ adsorbents that will effectively capture CO_2_ from the atmosphere. These changes will be possible to slow down, stop, and even reverse global warming and many unions and countries around the world are contributing to this goal. The European Union (EU) could be mentioned, which has committed all EU members to a 55% reduction in CO_2_ emissions by 2030 and carbon neutrality by 2050^1^. The U.S. The Department of Energy (DOE) has set an ambitious target of 90% capture of the CO_2_ from natural gas flue streams^2^ and many more.

Another way to reduce CO_2_ emissions is to transform fossil fuel combustion transport as one of the primary producers of CO_2_ into alternative energy sources. There are currently two options to choose: electric motors or hydrogen-powered engines. Although hydrogen engines' design, technology, and performance are at a high level, the problem is insufficient vehicle mileage. For this reason, there is a need to develop fuel tanks that can store large amounts of hydrogen. The U.S. Department of Energy (DOE) has set 2025 technical targets for hydrogen gravimetric capacity of 5.5 wt.% H_2_, operating in the range of 40/85 °C^3^.

In both above-discussed applications, hybrid inorganic–organic materials called metal–organic frameworks (MOFs) are intensively studied and developed. MOFs belong to a class of coordination compounds composed of metal cations/clusters connected by organic linkers to form porous three-dimensional polymeric frameworks. Large surface area, different pore shapes/sizes, and pore volumes can be effectively tuned based on appropriately selected building blocks and their compatibility. Another advantage is the post-synthetic modifiability of frameworks which can increase the efficiency of the material in the selected application. MOFs find their usage in gas adsorption and separation^4–7^, heterogeneous catalysis^8,9^, sensors^10–12^, magnetic refrigeration^13,14^, drug delivery^15,16^, or as SARS-CoV-2 detection and elimination materials^17,18^. In CO_2_ storage of unmodified MOF materials, the leaders at low pressures are: Mg-MOF-74 (30.1 wt.% @ 20 °C and 1 bar)^19^, Mg_2_(dobpdc) (23.8 wt.% @ 40 °C and 1 bar)^20^ and at high pressures UiO(bpdc) (79.7 wt.% @ 30 °C and 20 bar)^19^, NU-111 (61.8 wt.% @ 20 °C and 30 bar)^21^. Post-synthetic modification can increase the stored amount of CO_2_^22^, such as tetraethylenepentamine-modified Mg-MOF-74 whose initial capacity of 23.4 wt.% has increased to 26.9 wt.% (@ 20 °C and 1 bar)^23^, or polyethylenimine-modified MIL-101, 1.32 wt.% → 18.48 wt.% (@ 20 °C and 0.15 bar)^24^. Hydrogen storage capacities in the top MOF materials @ − 196 °C and 1 bar ranged in 2.0–2.8 wt.%^25,26^ and at high pressures the best H_2_ adsorbents include: DUT-32 (14.2 wt.% @ − 196 °C and 82 bar)^27^, she-MOF-1 (12.6 wt. % @ − 196 °C and 100 bar)^28^ and SNU-77H (11.0 wt.% @ − 196 °C and 90 bar) ^29^.

In the present study, we were inspired by amine-functionalized silicas with which our working group has many years of experience^30,31^. We applied a similar approach for MOF material (HKUST-1), which was post-synthetically modified with diamine (ethylenediamine, *en*) and triamine (diethylenetriamine, *deta*) on coordinatively unsaturated sites (CUSs) within HKUST-1 framework. Amines are known to have a high ability to capture CO_2_ molecules, and therefore HKUST-1 has been functionalized with mentioned amines in different molar ratios. It is known that the calculated ideal pore size of material for efficient H_2_ storage is 6–7 Å^32,33^. We modified HKUST-1 by varying the concentration of amines to achieve maximum H_2_ storage. The prepared materials were subsequently studied as CO_2_ and H_2_ adsorbents at 0 °C and − 196 °C, respectively. It was shown that the enhanced capacity of the materials towards the selected adsorptives increased with an decreasing amine molar ratio. In addition, it can be concluded that better results were observed for *deta*-modified materials compared to *en*-functionalized materials.

## Experimental

### Chemicals, synthesis and post-synthetic modification

All chemicals used to synthesise HKUST-1 and its subsequent post-synthetic modification were purchased in the highest available purity from Acros Organics and Sigma Aldrich.

#### HKUST-1

1 g (4.76 mmol) of benzene-1,3,5-tricarboxylic acid (H_3_BTC) was dissolved in a 30 ml solvent mixture of ethanol and *N, N′*-dimethylformamide (DMF) (1:1, v:v). Subsequently, 15 ml of an aqueous solution of copper nitrate trihydrate (2.08 g, 8.6 mmol) was added to the H_3_BTC solution. The mixture was stirred for 10 min and finally allowed to react in an oven at 85 °C for 24 h. After a mentioned time, the resulting product was filtered off and dried. The weight of the prepared HKUST-1 was 2.088 g. Further, DMF (b.p. 153 °C) molecules located in the cavities of as-synthesized (AS) HKUST-1 were exchanged (EX) for ethanol (b.p. 78 °C) for subsequent easier activation at lower temperatures to obtain HKUST-1 in activated form (AC). The solvent exchange process was performed in a Soxshlet extractor for 48 h.

#### Post-synthetic modification

100 mg of activated HKUST-1 (200 °C, 30 min) was dispersed in 10 ml of dry methanol under a nitrogen atmosphere. A 0.5 M solution of amine (diethylamine (*en*), diethylenetriamine (*deta*)) in dry methanol was added to the suspension in various proportions. The selected molar ratios for Cu(II) in HKUST-1: amine were 1:2; 1:1.5; 1:1; 1:0.5; 1:0.25; 1:0.1 for *en* and 1:2; 1:1.5; 1:1; 1:0.75; 1:0.5; 1:0.25; 1:0.1; 1:0.05 for *deta*. The reaction mixtures were stirred for 24 h and after the reaction time, the amine-modified products were filtered, several times washed with methanol, dried, and further analyzed.

### Methods and characterization

Infrared spectra (IR) of the prepared materials were measured on a Nicolet 6700 instrument in the wavelength range of 4000–400 cm^−1^ using the ATR method. Elemental analysis was measured on a CHNOS Elemental Analyzer Vario MICRO instrument with a sample weight of ~ 2 mg. Thermoanalytical experiments, thermogravimetry with simultaneous different scanning calorimetry coupled with mass spectrometry (TG/DSC-MS) were performed using a SetsysEvolution (Setaram). The measurements were carried out with 16–19 mg of the sample using α-Al_2_O_3_ crucible. TG-DSC curves were recorded in the inert atmosphere of Ar (20 cm^3^ min^−1^) from 15–800 °C with a heating rate of 10 °C min^−1^. Thermal analysis with simultaneous differential thermal analysis (TG/DTA) was carried out on a Netzsch STA 449 F1 Jupiter. The sample with the weight of ~ 20 mg was placed in a corundum crucible and heated from 30 to 900 °C with a heating rate of 10 °C min^−1^ in the air atmosphere with a flow rate of 60 cm^3^ min^−1^. Powder X-ray diffraction (PXRD) experiments were done in reflection geometry on a Rigaku MiniFlex 600 multipurpose diffractometer using Cu/Kα radiation (*λ* = 1.54056 Å) and 2*θ* continuous scan at 2° min^−1^ from 2 to 60°. Adsorption and desorption of Ar, CO_2_ and H_2_ were performed at − 186 °C, 0 °C, and − 196 °C, respectively, using the Autosorb iQ-XR (Quantachrome Instruments). Void volume was determined by a helium-free NOVA^®^ approach^34^. Samples were activated in a vacuum (20 h, 150 °C). The volume of micropores (*V*_*p, micro*_, pore diameter < 2 nm) and mesopores (*V*_*p, meso*_, pore diameter 2–50 nm) were calculated from pore size distribution curves obtained by fitting the Ar adsorption data by a Non-Local Density Functional Theory (NLDFT) adsorption kernel (ASiQwin software, Quantachrome Instruments) assuming cylindrical pores. BET area (*S*_*BET*_) was calculated according to the procedure for microporous materials^35^, based on which adsorption isotherm points in the region of ca. 10^−3^–10^−2^ of *p/p*_*0*_ (relative pressure) were used; 0.142 nm^2^ as a cross-sectional area of Ar molecule was assumed.

## Results and discussion

### HKUST-1 structure and synthesis

**Figure 1:**
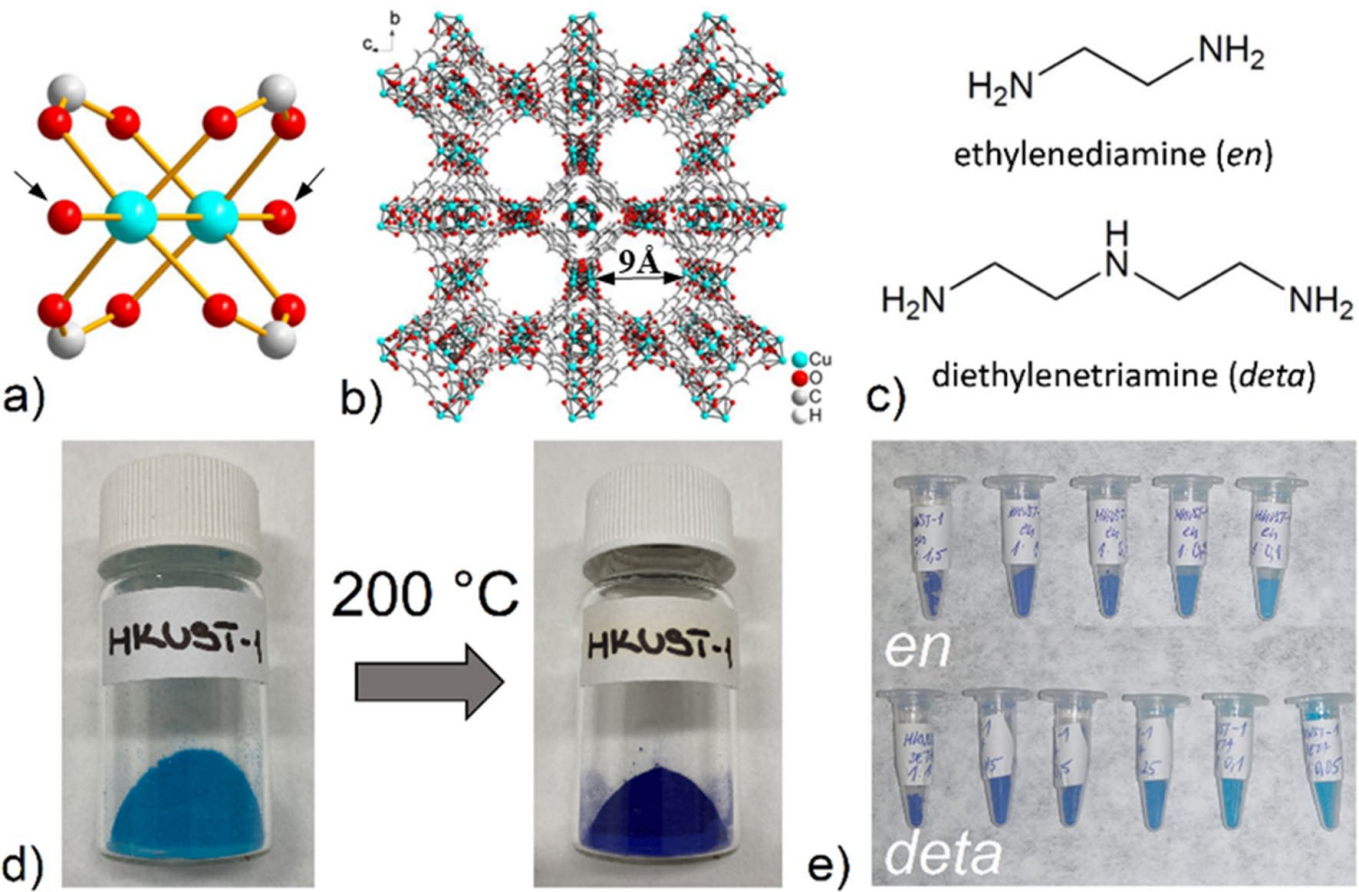
(**a**) Paddle-wheel cluster, where the arrows indicate the oxygen of the coordinated water molecules and (**b**) final framework of HKUST-1^36^. (**c**) Molecular structure of amines (*en* and *deta*) used in post-synthetic modification. (**d**) Solvatochromism present in activated HKUST-1 at 200 °C. (**e**) A view of the prepared materials and their different colors depending on the molar ratio of amines used.

HKUST-1 is one of the first MOF compounds, which attract scientists around the world in the preparation and application of metal–organic structures^36^. The crystal structure of HKUST-1 consists of two copper(II) cations with coordination number five, which form a paddle-wheel cluster with a square secondary building unit (SBU). Four carboxylate groups are coordinated to the cluster in *syn-syn* mode, forming a tetragonal base, and the fifth Cu(II) coordination position is occupied by coordinated water molecules to form a tetragonal pyramid-shaped polyhedron, as shown in Fig. 1a. The mutual combination of the mentioned building blocks results in the final 3D polymeric framework of HKUST-1 with bimodal pore size distribution, small cage with diameter 3.5 Å and large cage with diameter 9 Å (see Fig. 1b). The post-synthetic modification of HKUST-1 consisted of replacing coordinated water molecules in the paddle-cluster with amine molecules with different numbers of amine functional groups (ethylenediamine (*en*, diamine) and diethylenetriamine (*deta*, triamine)), see Fig. 1c. Water molecules were removed by heating the compound at 200 °C to form coordinatively unsaturated sites (CUSs) to which amines were attached via nitrogen donor atoms. Activation of the material was accompanied by a color change (from blue to purple, see Fig. 1d) due to solvatochromism, which is caused by a change in the donor set and coordination environment of the central atoms. The selected molar ratios for Cu(II) in HKUST-1: *en* were 1:2; 1:1.5; 1:1; 1:0.5; 1:0.25; 1:0.1 and for Cu(II) in HKUST-1: *deta* were 1:2; 1:1.5; 1:1; 1:0.75; 1:0.5; 1:0.25; 1:0.1;1:0.05. At high molar ratios (= high amine concentrations), HKUST-1 was decomposed due to a strongly alkaline environment. In the case of *en*, the decomposition of HKUST-1 occurred at a molar ratio of 1:2 and *deta* at ratios of 1:2 and 1:1.5. At medium ratios (1:1.5; 1:1 for *en* and 1:1; 1:0.75;1:0.5 for *deta*) there was no decomposition of HKUST-1, but its color change (from blue to violet) was observed. At low amine concentrations (1:0.1; 1:0.25; 1:0.5 for *en* and 1:0.05; 1:0.1; 1:0.25 for *deta*) the materials retained the original color as the original HKUST-1, as depicted in Fig. 1e.

### Characterization

Infrared spectroscopy (IR), thermal analysis (TG / DSC) and powder X-ray diffraction (PXRD) methods were used to identify HKUST-1, monitor the post-synthetic modification process, thermal stability and characterization of prepared materials.

**Figure 2:**
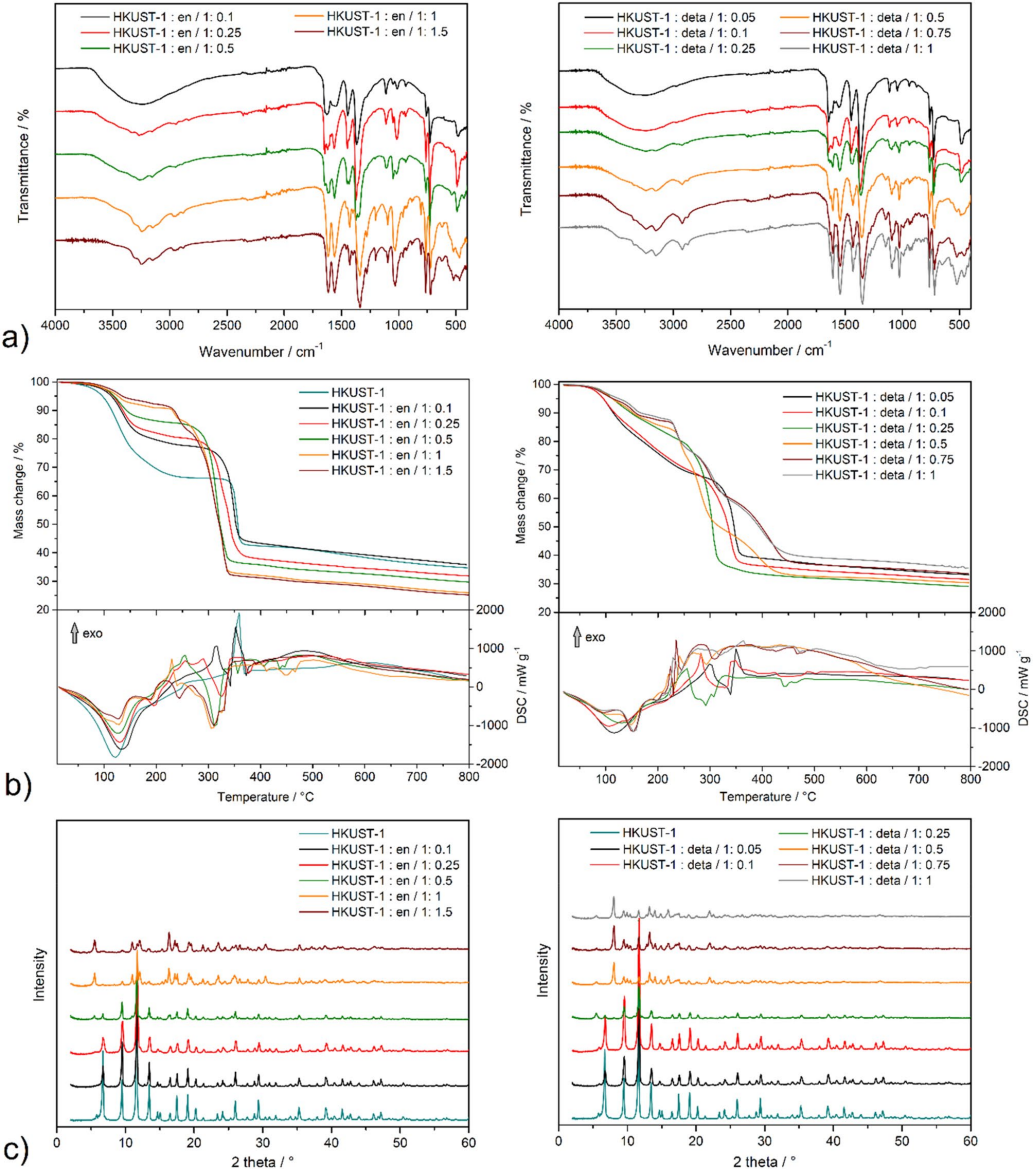
(**a**) Infrared spectra of HKUST-1 materials modified with *en* and *deta*. (**b**) TG/DSC curves of prepared materials measured in the temperature range of 20–800 °C. (**c**) PXRD patterns of prepared materials in the 2 theta range of 2–60°.

The IR spectra of the materials are shown in Fig. 2a, Fig. S1 in ESI and the assignment of the characteristic vibration absorption bands are summarized in Table 1 in ESI. The comparison of IR spectra of as-synthesized (AS), solvent exchanged (EX) and activated (AC) HKUST-1 is shown in Fig. S1 in ESI and confirm successful ethanol exchange process and material’s activation based on the presence/absence of characteristic solvent absorption bands. In all infrared spectra (see Fig. 2a), a broad absorption band at 3400 cm^−1^ is present, which can be assigned to the OH stretching vibration (*ν*(OH)) of the physisorbed water molecules. BTC linker in the materials is evident due to the presence of several characteristic vibrations: *ν*(COO^−^)_as_ about 1550 cm^−1^, *ν*(COO^−^)_s_ in the range of 1340–1370 cm^−1^, aromatic *δ*(CCH)_ar_ around 1100 cm^−1^ and *δ*(COO^−^) about 720 cm^−1^. For the modified materials, a gradual increase in intensity of *ν*(NH) around 3200 cm^−1^, *δ*(NH) about 1580 cm^−1^, several absorption bands of aliphatic *ν*(CH)_aliph_ under 3000 cm^−1^ can be observed in the IR spectra with increasing concentration of amines, which are characteristic for *en* and *deta* (see Fig. 2a, Table 1 in ESI).

**Table 1:** Obtained and calculated results of textural properties determined from Ar adsorption measurements @ − 186 °C and storage capacity of CO_2_ and H_2_ measured 0 °C and − 196 °C, respectively of prepared materials in different units.

Material	Ar @ − 186 °C			CO_2_ @ 0 °C			H_2_ @ − 196 °C		
	*S*_BET_ (m^2^ g^−1^)	*V*_*p, micro*_ (cm^3^ g^−1^)	*V*_*p, meso*_ (cm^3^ g^−1^)	*V *(cm^3^ g^−1^)	*w* (wt.%)	*n* (mmol g^–1^)	*V* (cm^3^ g^−1^)	*W* (wt.%)	*n* (mmol g^−1^)
HKUST-1	1468	0.681	0.000	77.3	15.19	3.45	165.3	1.49	7.37
HKUST-1: *en*/1:0.1	656	0.321	0.063	113.6	22.31	5.07	202.1	1.82	9.02
HKUST-1: *en*/1:0.25	511	0.251	0.067	88.8	17.45	3.96	155.7	1.40	6.95
HKUST-1: *en*/1:0.5	309	0.147	0.068	46.7	9.18	2.09	82.9	0.75	3.70
HKUST-1: *en*/1:1	53	0.025	0.007	11.4	2.24	0.51	11.3	0.10	0.50
HKUST-1: *en*/1:1.5	–	–	–	3.1	0.61	0.14	6.5	0.06	0.29
HKUST-1: *deta*/1:0.05	1439	0.671	0.034	168.4	33.09	7.52	253.8	2.28	11.33
HKUST-1: *deta*/1:0.1	1245	0.577	0.050	156.7	30.79	7.00	236.4	2.12	10.55
HKUST-1: *deta*/1:0.25	769	0.377	0.034	93.5	18.37	4.17	156.9	1.41	7.00
HKUST-1: *deta*/1:0.5	311	0.146	0.011	37.7	7.41	1.68	67.9	0.61	3.03
HKUST-1: *deta*/1:0.75	90	0.043	0.010	14.0	2.74	0.62	44.0	0.40	1.96
HKUST-1: *deta*/1:1	7.2	–	–	5.4	1.06	0.24	36.9	0.33	1.65

*S*_BET_ BET area, *V*_*p*_ pore volume, *V* adsorbed gas volume, *w* mass percent, *n* amount of substance, - undetecable, micro micropore, meso mesopore.

Thermal robustness and behavior of prepared materials were investigated by a combination of thermogravimetric (TG) analysis and differential scanning calorimetry (DSC) (see Fig. 2b). Unmodified HKUST-1 is thermally stable up to 50 °C, above mentioned temperature the compound desolvates in the temperature range 50–250 °C with a weight loss of 33.5 wt.% on TG. The activated form is stable up to 330 °C, as it seems by the plateau on TG curve, and subsequently, in the interval 330–380 °C thermal decomposition of the framework occurs with a mass change of 23.2 wt.%. For *en*-modified materials in a molar ratio of 1:0.1 to 1:0.5, the shape and course of the TG curves are identical to unmodified HKUST-1, and the thermal decomposition takes place in two steps (see Fig. 2b, left). From the TG curves of mentioned materials, it is evident that they contain less amount of solvents in the cavities (15.2–22.6 wt.%, range 20–265 °C), and higher weight loss is observed in the thermolysis of the frameworks (33.6–48.5 wt.%, range 265–385 °C) compared to HKUST-1. This observation can be explained by the increasing content of *en* molecules in materials, which reduce the free void volume for solvents and increase the content of organic building blocks in the compounds. Several changes on the TG curves of HKUST-1: *en*/1:1 and HKUST-1: *en*/1:1.5 are observed: smaller amounts of solvents (8.3–9.4 wt.%, range 20–220 °C), thermal decomposition takes place in three steps, higher content of organic components (57.9–59.8 wt.%) and reduced thermal stability of the organic part (220–350 °C). Regarding the thermal stability of *en*-modified materials, it should be noted that they show lower thermal stability of the framework compared to HKUST-1. Similar thermal behavior was observed for the *deta*-modified materials as for the *en*-functionalized samples (see Fig. 2b, right). The course of TG curves HKUST-1: *deta* with molar ratios of 1:0.05, 1:0.1 and 1:0.25 show a similar trend as unmodified HKUST-1 (desolvation 20.5–30.6 wt.%, range 20–250 °C and organic part 29.9–45.6 wt.%, range 250–370 °C). Increasing the concentration of *deta* is associated with the course changes of the TG curves on which five decomposition steps are observed and indicate a significant chemical change of the prepared materials. In general for all thermogravimetric curves, in the first thermal decomposition steps, desolvation/dehydration occurred in the mass change interval of 8.1–34.2 wt.% on TG curves, which are accompanied by endothermic effects on DSC curves with peaks in the range of 96–154 °C. Pyrolysis of the organic moiety occurred in the 220–450 °C temperature range, which is followed by exothermic effects on the DSC in the range of 220–370 °C. According to the changes in TG curves and colour change of materials with higher amine content, we assumed a phase change of these compounds, which was confirmed by PXRD measurements (see text below).

The structural and thermal stability of HKUST-1 and materials after functionalization with *en* and *deta* in different molar ratios was monitored by PXRD measurements (see Fig. 2c, Figs. S2–S5 in ESI). In Fig. S2 in ESI the comparison of measured and calculated PXRD (Chui et al. 1999) patterns of HKUST-1 is presented, which are in good agreement and confirm the successful preparation of HKUST-1. Heating PXRD measurements studied the thermal stability of HKUST-1 at 50, 100, 150, 200, 250 and 350 °C (see Fig. S3 in ESI), which showed that HKUST-1 is stable after the activation process and its decomposition occurs at 350 °C. The obtained results are in good agreement with TG measurements (see Fig. 2b). From the PXRD patterns of HKUST-1: *en* materials (see Fig. 2c, left), it is evident that at molar ratios of 1:0.1 and 1:0.25 only the HKUST-1 phase is present. At a ratio of 1:0.5, in addition to the diffraction peaks corresponding to HKUST-1, novel diffraction lines appear, corresponding to the formation of a new phase. A further increase in the concentration of *en* completely eliminates HKUST-1, while only the diffraction peaks of the novel phase are present in the PXRD patterns. Similar changes were observed for *deta*-modified materials (see Fig. 2c, right). The HKUST-1 phase is stable up to a molar ratio of 1:0.1, at a ratio of 1:0.25, a mixture consisting of HKUST-1 and a new phase is present, and at higher molar ratios, only a novel phase is present. The described phase changes were manifested visually by a colour change of prepared materials from blue to purple (see Fig. 1e). The thermal stability of selected *en*-modified materials at a low ratio, HKUST-1: *en*/1:0.25 represented by HKUST-1 phase (see Fig. S4 in ESI) and a high ratio, HKUST-1: *en*/1:1 characterised by novel phase (see Fig. S5 in ESI) was also studied. For the low ratio material, the same thermal stability as pure HKUST-1 was observed, and the high molar sample is thermally stable up to 100 °C, at higher temperatures, a phase change occurs, and at 350 °C the material decomposed.

**Figure 3:**
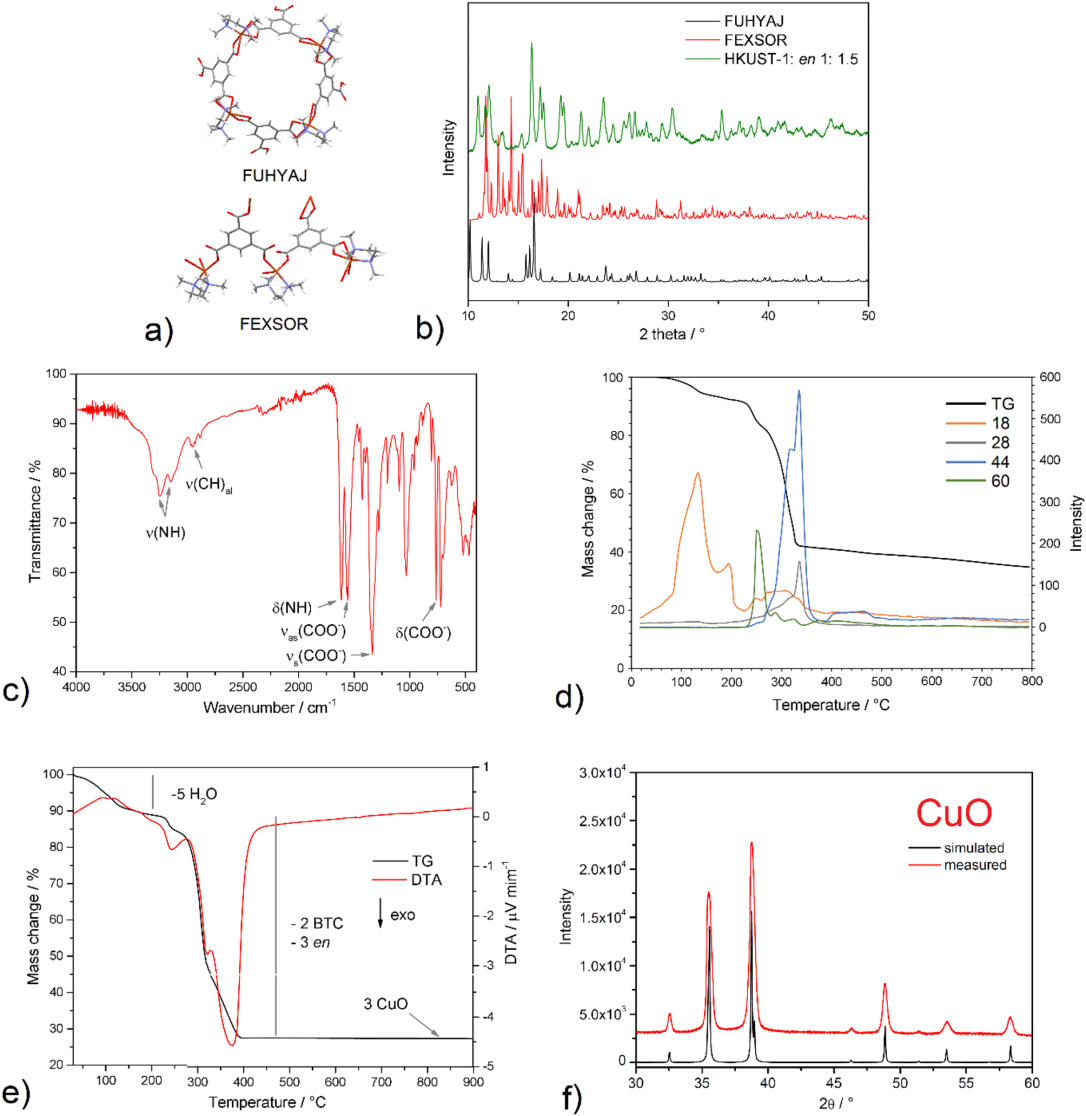
(**a**) The crystal structure of Cu_4_(HBTC)_4_(*tmen*)_4_·12H_2_O (FUHYAJ) and Cu_3_(BTC)_2_(*tmen*)_3_(H_2_O)_2_·6.5H_2_O (FEXSOR). (**b**) Comparison of measured PXRD pattern of sample HKUST-1: *en*/1:1.5 and calculated patterns of Cu_4_(HBTC)_4_(*tmen*)_4_·12H_2_O (FUHYAJ) and Cu_3_(BTC)_2_(*tmen*)_3_(H_2_O)_2_·6.5H_2_O (FEXSOR). The detailed characterisation of HKUST-1: *en*/1:1.5 using (**c**) infrared spectroscopy, (**d**) TG-MS in an argon atmosphere, (**e**) TG/DTA in an air atmosphere. (**f**) Identification of final thermal decomposition product by PXRD.

The new phase that forms at high amine concentrations (specifically HKUST-1: *en*/1:1.5) was studied through various physicochemical techniques in order to identify it and determine its chemical composition. By searching the CCDC (Cambridge Crystallographic Data Center) database, it can be concluded that two compounds containing Cu(II), BTC(-III) ions and a derivative of *en* ligand with the chemical composition Cu_4_(HBTC)_4_(*tmen*)_4_.12H_2_O (FUHYAJ^37^) and Cu_3_(BTC)_2_(*tmen*)_3_(H_2_O)_2_·6.5H_2_O (FEXSOR^38^) were synthesized (see Fig. 3a). In their preparation, a *N*-tetramethyl-substituted *en* ligand (*tmen—N,N,Nʹ,Nʹ*-tetramethylethylenediamine) on nitrogen atoms was used. By comparing the PXRD pattern of HKUST-1: *en*/1:1.5 material and the mentioned compounds, a similarity with the compound Cu_3_(BTC)_2_(*tmen*)_3_(H_2_O)_2_·6.5H_2_O can be observed (see Fig. 3b). Since it was not possible to clearly identify the newly formed phase by the PXRD comparison, we decided to study the exact chemical composition of the modified material HKUST-1: *en*/1:1.5. The presence of organic building blocks (BTC and *en*) was confirmed by IR spectroscopy. The IR spectrum (Fig. 3c) of compound contains characteristic absorption bands for the carboxylate group of the BTC linker (*ν*(COO^−^)_as_ 1563 cm^−1^, *ν*(COO^−^)_as_ 1337 cm^−1^ and *δ*(COO^−^) 723 cm^−1^) and the presence of *en* is confirmed by typical bands for aliphatic CH_2_ (*ν*(CH)_al_ 2970, 2947, 2927 and 2884 cm^−1^) and NH_2_ groups (*ν*(NH) 3248 and 3148 cm^−1^; *δ*(NH) 1615 cm^−1^). By TG-MS, the thermal stability of the new phase was studied in an inert atmosphere of argon, through which the presence of *en* was also confirmed (see Fig. 3d). In the temperature interval 60–220 °C, the release of water molecules (dehydration) occurs, which was manifested on the MS spectrum by the signal *m/z* = 18. In the second step of thermal decomposition in the temperature interval 220–280 °C, a mass loss is observed, which corresponds to the release of coordinated *en* molecules with a signal of *m/z* = 60. Further heating in the temperature range of 280–350 °C results in pyrolysis of the polymer skeleton and further release of *en*. The mass spectra of gaseous products in the mentioned interval showed the signals with *m/z* = 18, 32, 44, 60 corresponding to H_2_O, CO, CO_2_ and *en*, respectively. The above results show that the thermal decomposition of *en* and BTC cannot be clearly distinguished, and both decomposition steps take place simultaneously. As the thermal analysis was carried out in an inert atmosphere of argon, the sample did not fully decompose, and carbonized material was present in the resulting thermal decomposition product. For this reason, TG/DTA measurements were carried out in an oxidizing air atmosphere to ensure complete thermolysis of the material, and based on weight losses and CHN elemental analysis results, it was possible to determine the exact chemical composition of the sample HKUST-1: *en*/1:1.5. TG/DTA curves of HKUST-1: *en*/1:1.5 measured in the air atmosphere are shown in Fig. 3e. In the first thermal decomposition step, in the temperature interval 40–150 °C, a mass loss of 10.18% was observed, which corresponds to the release of five water molecules (clcd. mass change 10.29%). Dehydration of the sample was manifested on the DTA curve by two endothermic effects at 96 and 118 °C. In the following two overlapping decomposition steps, the release of three amine molecules and the thermal decomposition of two BTC molecules occur. The total mass loss corresponding to the organic components was 61.77% (clcd. mass change 62.45%), and their decomposition/release was accompanied by three exothermic effects on the DTA curve at 244, 324 and 377 °C. PXRD identified the resulting thermal decomposition product as CuO (see PXRD pattern in Fig. 3f) with the experimental residual mass of 27.94% (clcd. residual mass 27.27%). Based on the described weight losses, it was possible to determine the exact chemical composition of the material HKUST-1: *en*/1:1.5 with the formula Cu_3_(BTC)_2_(*en*)_3_·5H_2_O. The chemical composition was also confirmed by CHN elemental analysis, while the calculated and measured percentages of chemical elements in the compound are in good agreement (*M*_*r*_ = 875.25 g mol^−1^; exp.: C 32.54%, H 4.51%, N 21.92%, clcd.: C 32.93%, H 4.61%, N 21.78%).

### Texture and gas adsorption properties

**Figure 4:**
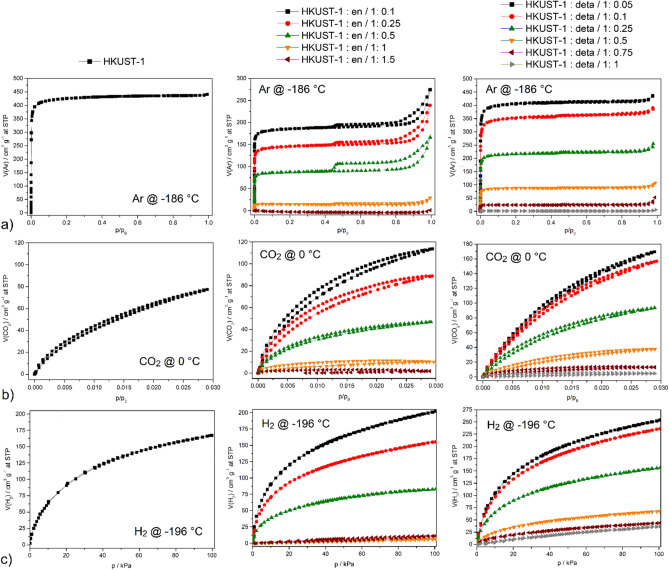
Adsorption/desorption isotherms of (**a**) argon @ − 186 °C, (**b**) carbon dioxide @ 0 °C and (**c**) hydrogen @ − 196 °C on pristine HKUST-1, *en* and *deta*-modified HKUST-1 materials.

After successful preparation and characterization of the amine-modified compounds, the materials’ textural properties (*S*_*BET*_ area, micropore and mesopores volume) were studied by argon adsorption @ − 186 °C, and their storage capacities of carbon dioxide @ 0 °C and hydrogen @ − 196 °C were investigated. The results obtained from the adsorption measurements are shown in Fig. 4 and summarized in Table 1.

Argon adsorption measurements @ − 186 °C studied the effect of post-synthetic amine-modification on the textural properties of HKUST-1. Figure 4a shows a comparison of the Ar adsorption/desorption isotherms of pristine HKUST-1, *en*- and *deta*-modified materials, and the selected textural properties calculated from the respective isotherms are summarized in Table 1. The materials show a typical type *Ia* isotherm classified by IUPAC^35^, characteristic for materials with narrow micropores below ca. 1 nm in diameter. This is in line with obtained micropore sizes that were the same for all studied materials and ranged from 0.6 to 1.2 nm. As can be seen from the obtained results, the *S*_*BET*_ area of pristine HKUST-1 was 1468 m^2^ g^−1^, and after modification, the area of materials decreases with increasing amine concentration. The *S*_*BET*_ area of modified materials decreased from HKUST-1: *en*/1:0.1 (656 m^2^ g^−1^) to undetecable values for HKUST-1 : *en*/1:1.5 for *en*-modified samples, and in *deta*-functionalized materials from HKUST-1: *deta*/1:0.05 (1439 m^2^ g^−1^) to undetecable values for HKUST-1: *deta*/1:1. The observed phenomenon can be explained by the reduction in the free pore volume/area of the modified materials due to the binding of a larger number of molecules and the bulk of amine molecules themselves. It can be seen that even a low molar ratio of *en* (1:0.1) in HKUST-1 structure leads to a halving of textural properties like *S*_*BET*_. On the contrary, the same content of *deta* leads to only a small decrease in texture parameters (1245 m^2^ g^−1^). The observed difference can be explained based on the length of amines used and their coordination modes to coordinatively unsaturated sites (CUSs) within the HKUST-1 framework. The size of *en* molecule is 3.7 Å, and *deta* molecule is 6.3 Å (see Fig. S6a in ESI). Since the smallest distance between two Cu(II) ions within the HKUST-1 skeleton is 5.2 Å (see Fig. S6b in ESI), *deta* molecules can bridge and connect the two metal centres, while *en* can only bind terminally. The mentioned coordination modes of amine molecules are schematically drawn in Fig. S6c in ESI. Fig. S6c in EIS shows the entrance window to the HKUST-1 porous framework, where four Cu(II) cations are located inside the pore. When amines are coordinated to the metal centre, four molecules of *en* or two molecules of *deta* are coordinated within the entrance window, as shown in Fig. S6c in ESI. Thus, in the case of *en* coordination, the free pore volume is more efficiently filled, resulting in a lower surface area than bulkier *deta* molecules. To confirm the assumed theory, we prepared HKUST-1 modified material with *bapen* (*bapen* = *N,N′*-bis(3-aminopropyl)-1,2-diaminoethane). *Bapen* is a dimensionally longer ligand compared to *en* and *deta* and contains four amine groups connected by ethylene and propylene bridges (see inset in Fig. S7 in ESI). By comparing the surface area of modified HKUST-1 materials: HKUST-1: *en*/1:0.1 (656 m^2^ g^-1^) < HKUST-1: *bapen*/1:0.1 (1028 m^2^ g^−1^) < HKUST-1: *deta*/1:0.1 (1245 m^2^ g^−1^), it can be concluded that in material HKUST-1: *bapen*/1:0.1 extended amine ligand also did not cause a significant decrease in *S*_*BET*_ as in the case of *en* (see Fig. S7 in ESI).

When reducing the textural properties of materials due to the increase in the concentration of amines, it is also necessary to consider the results of PXRD analysis. PXRD measurements revealed the formation of a new phase, which is present in the mixture at medium molar ratios and in pure form at the highest ratios. As is evident from the measured and calculated textural properties, the newly formed phase is non-porous, as the materials HKUST-1: *en*/1:1, HKUST-1: *en*/1:1.5, HKUST-1: *deta*/1:0.75 and HKUST-1: *deta*/1:0.75 adsorb only minimally amounts of argon. Moreover, on argon adsorption isotherms an increase in the Ar adsorbed volume at higher relative pressures is observed, as well as H4 hysteresis loop typical for aggregated crystals^35^ indicating the presence of mesopores especially in the *en-*modified materials with ratios from 1:0.1 to 1:0.5 containing low concentration of *en*. Presence of mesopores or larger pores can be explained by formation of intraggregate voids^39^ due to surface modification, or etching of the material caused by increasing the basicity of the environment during the modification process. As shown in Table 1, the percentage increase in mesopore volume (*V*_*p, micro*_/*V*_*p, meso*_) rises with increasing amine concentration, and the values increase from 20 to 46% for HKUST-1: *en* and from 5 to 23% for HKUST-1: *deta*. Similar changes in textural properties were observed for other amine-modified MOF materials such as MOF-74^23^, MOF-177^39^ and IRMOF-74^40^.

From the measured carbon dioxide adsorption/desorption isotherms, an increasing amount of stored CO_2_ can be generally observed with the decreasing concentration of amines (see Fig. 4b, Table 1). The pristine HKUST-1 material can store in its framework 15.19 wt.% (3.45 mmol g^−1^) of CO_2_ @ 0 °C and 1 bar. The maximum CO_2_ storage capacity of *en*-modified materials was measured on HKUST-1: *en*/1:0.1 with a value of 22.31 wt.% (5.07 mmol g^−1^) and decreased to 0.61 wt.% (0.14 mmol g^−1^) for HKUST-1: *en*/1:1.5 @ 0 °C and 1 bar. For *deta*-modified materials, the highest CO_2_ uptake of 33.09 wt.% (7.52 mmol g^−1^) was obtained for HKUST-1 : *deta*/1:0.05 and the lowest value of 1.06 wt.% (0.24 mmol g^−1^) for HKUST-1 : *deta*/1:1 @ 0 °C and 1 bar. In the case of CO_2_ adsorption, it is necessary to consider the competitive processes of chemisorption carried out on amine groups (R–NH_2_+CO_2_=R–NH_2_COO) and physisorption, which takes place in the void pore volume of materials. As already discussed in the context of Fig. S6, the void volume seems to be larger for materials modified by *deta,* than *en*, which is in line with the fact that *deta*-modified material (e.g. 1:0.1) exhibits larger CO_2_ adsorption capacity compared to material modified by an equivalent quantity of *en*. In terms of concentration, although the number of active sites for the sorption of CO_2_ molecules increases with the increasing concentration of coordinated amines, the free pore volume within the framework decreases due to the bulkiness of amines. The phenomenon described is clear from the measured results of CO_2_ adsorption for amine-modified materials (see Fig. 4b, Table 1). Another fact that needs to be considered is the already mentioned phase change of materials at higher amine molar ratios, leading to non-porous materials’ formation. A trend of decreasing CO_2_ adsorption capacity with increasing amine concentration was also observed for MOF-177 functionalized with polyethyleneimine (PEI) and *deta*^39^. CO_2_ storage capacity decreased for PEI modified materials in following order: MOF-177-PEI (10%) (12.8 wt.%) > MOF-177-PEI (20%) (10.6 wt.%) > MOF-177-PEI (30%) (9.7 wt.%) and *deta*-modified materials in the order: MOF-177-*deta* (20%) (12.3 wt.%) > MOF-177-*deta* (30%) (9.6 wt.%) @ 25 °C and 1 bar. The UiO-66 material modified with PEI could also be mentioned, whose CO_2_ capacities decreased in the order of 11.9 wt.% for 20PEI@UiO-66 to 9.2 wt.% for 40PEI@UiO-66 @ 25 °C and 1 bar^41^. It can be summarized that the most promising material of our study with the highest adsorption capacity is HKUST-1: *deta*/1:0.05, which is able to adsorb 33.09 wt.% of CO_2_ (168.4 cm^3^ g^−1^, 7.52 mmol g^−1^).

Because hydrogen is a nonpolar and the smallest known molecule, the storage of large amounts of H_2_ is difficult, and for this reason, various MOF materials are intensively studied. It is known that efficient hydrogen storage can be achieved by the presence of alkali and alkaline earth metals within the framework, by the insertion of metal nanoparticles, or by post-synthetic modification^5,25,26^. Efficient hydrogen storage can also occur due to steric effects, as the calculated ideal pore size for hydrogen storage is 6–7 Å^32,33^. For this reason, the effect of different molar ratios of different bulk amines on the pore size in HKUST-1 was investigated to store H_2_ efficiently. The measured hydrogen adsorption isotherms of the prepared materials @ − 196 °C are shown in Fig. 4c, and the achieved H_2_ storage capacities in different units are listed in Table 1. As with Ar and CO_2_ adsorption, the same trend and thus increasing in adsorbed amounts of H_2_ with decreasing concentration of amines. The highest hydrogen uptakes were measured for HKUST-1 : *en*/1:0.1 and HKUST-1: *deta*/1:0.05 with 1.82 and 2.28 wt.%, respectively @ − 196 °C and 1 bar. The pore size can explain the increased affinity of H_2_ for the prepared materials. Calculated pore size distributions (PSD) were obtained by fitting the Ar adsorption data using a NLDFT adsorption kernel assuming cylindrical pores. All materials contain pore sizes ranging from 0.6 to 1.2 nm, covering the ideal pore size range of 0.6–0.7 nm. The decreasing H_2_ storage capacity of materials with increasing concentration of amines is due to the decreasing textural parameters like *S*_*BET*_ and *V*_*p, micro*_. The formation of a non-porous phase cannot be neglected in materials with a higher concentration of amines, through which it is possible to explain the decreasing H_2_ storage capacity of the compounds. According to the best of our knowledge, the effect of stored hydrogen on amine-modified MOFs has not yet been studied and the present study thus represents a pilot project. There are published articles dealing with the separation of H_2_/CO_2_ on amine-modified Mg-MOF-74 or MIL-53^42,43^, but not for H_2_ storage. In conclusion, however, it can be concluded that the measured highest storage capacities are in the range for top MOF materials^25,26^ and are comparable, for example, with UiO-66(Zr)^44^, MOF-519^45^, Mg-MOF-74^46^ with maximal H_2_ uptakes of 1.87 wt.%, 2.13 wt.% and 2.20 wt.%, respectively @ − 196 °C and 1 bar.

## Conclusion

The intention of present study was to increase the adsorption capacity of carbon dioxide and hydrogen in HKUST-1 through post-synthetic modification of amines with different numbers of amine groups. Ethylenediamine (*en*, diamine) and diethylenetriamine (*deta*, triamine) were chosen as amines in this study. At present, CO_2_ capture is realized using liquid amines, which can efficiently bind CO_2_ to form carbamates, but have many disadvantages (corrosivity, regeneration temperature). For this reason, a strategy of anchoring amines on porous support represented by the well-known MOF material, HKUST-1, was chosen. From the HKUST-1 stability point of view, it can be summarized that with increasing molar ratios of HKUST-1: amine, decomposition (1:2 for *en* and 1:2; 1:1.5 for *deta*), or a phase change (1:1.5; 1:1; 1:0.5 for *en* and 1:1; 1:0.75; 1:0.5; 1:0.25 for *deta*) were observed, which led to the formation of a non-porous materials, that have been thoroughly investigated. From the experimentally obtained adsorption measurements, it can be concluded that with the increasing concentration of amines, the CO_2_ capacity of materials decreased and that the *deta*-modified materials showed higher stored amounts of CO_2_ (probably due to their higher void volume) compared to *en*-modified compounds. The highest storage capacities were obtained for HKUST-1 : *en*/1:0.1 and HKUST-1 : *deta*/1:0.05 materials with 22.31 wt.% and 33.09 wt.% of CO_2_ @ 0 °C and 1 bar, respectively. The same trend was observed in hydrogen adsorption measurements, H_2_ storage capacity for HKUST-1: *en* 1:0.1 was 1.82 wt.% @ − 196 °C and 1 bar and for HKUST-1: *deta* 1:0.05 was 2.28 wt.% @ − 196 °C and 1 bar are comparable to other top MOF materials.

## Supplementary Information

Supplementary Information.

## Data Availability

The data are available on request, please contact corresponding author via e-mail (miroslav.almasi@upjs.sk).
