# Supplementary material for: Carbon dioxide and hydrogen adsorption study on surface-modified HKUST-1 with diamine/triamine
Source: Sci Rep. 2022 Oct 17;12:17366. doi: 10.1038/s41598-022-22273-2 (PMC9574841; doi:10.1038/s41598-022-22273-2)
Supplement: Supplementary file 1 — Supplementary Information. [file 41598_2022_22273_MOESM1_ESM.docx]

**Electronic Supplementary Information (ESI)**

**Carbon Dioxide and Hydrogen Adsorption Study on Surface-Modified HKUST-1 with Diamine/Triamine**

**Tomas Zelenka^1^, Klaudia Simanova^2^, Robin Saini^3^, Gabriela Zelenkova^1^, Satya Pal Nehra^4^, Anshu Sharma^3^, Miroslav Almasi^2*^**

^1^Department of Chemistry, Faculty of Science, University of Ostrava, 30. dubna 22, 701 03 Ostrava, Czech Republic

^2^Department of Inorganic Chemistry, Faculty of Science, P.J. Safarik University, Moyzesova 11, 040 01 Kosice, Slovak Republic

^3^Department of Physics, School of Engineering & Technology, Central University of Haryana, Mahendergarh 123031 India

^4^Center of Excellence for Energy and Environmental Studies, Deenbandhu Chhotu Ram University of Science and Technology, Murthal 131039, India

**Corresponding author: miroslav.almasi@upjs.sk*

**Figures:**


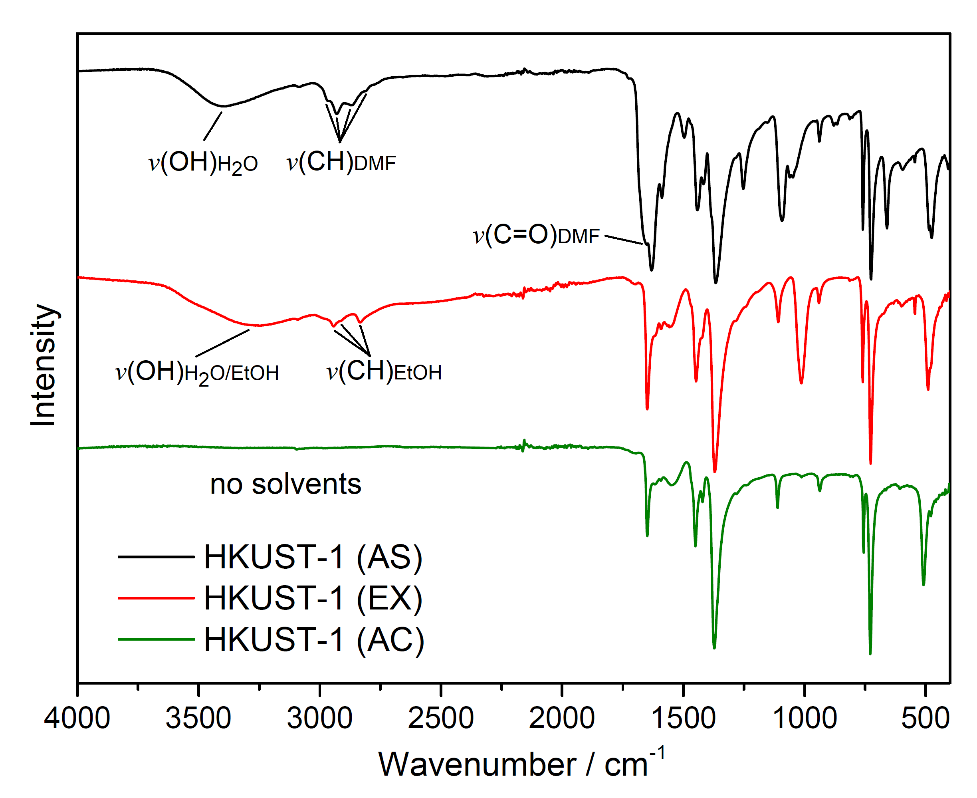


**Figure S1** Infrared spectra of as-synthesized (AS), ethanol exchanged (EX) and activated (AC) HKUST-1 with assignment of characteristic vibration to solvent molecules.


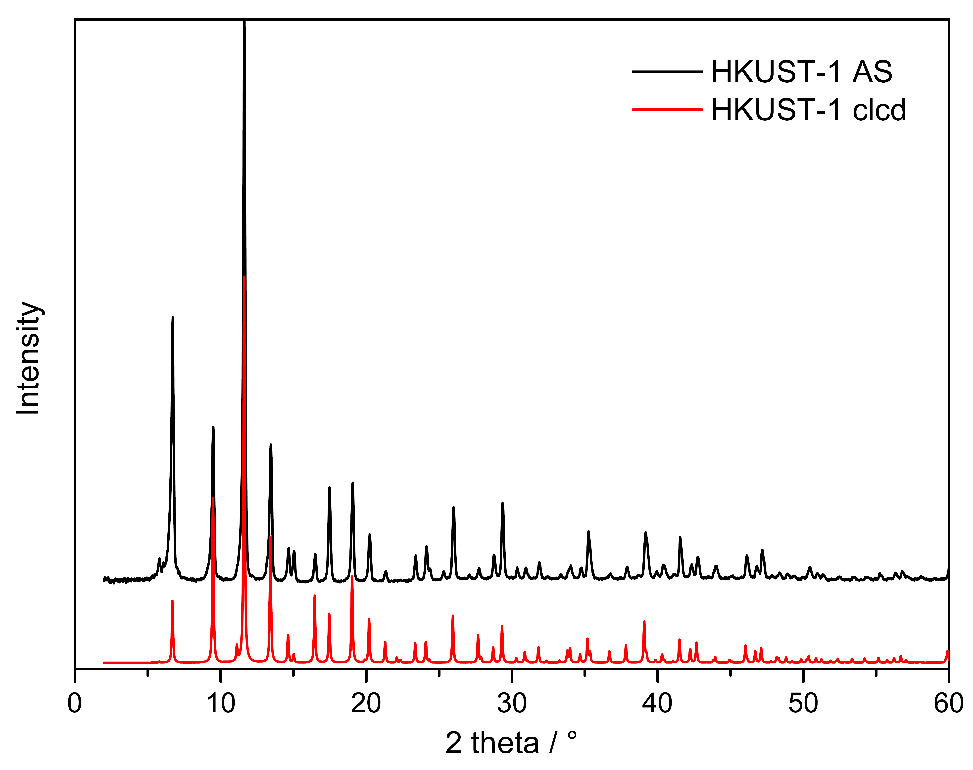


**Figure S2** Comparison of measured and calculated PXRD patterns of HKUST-1.


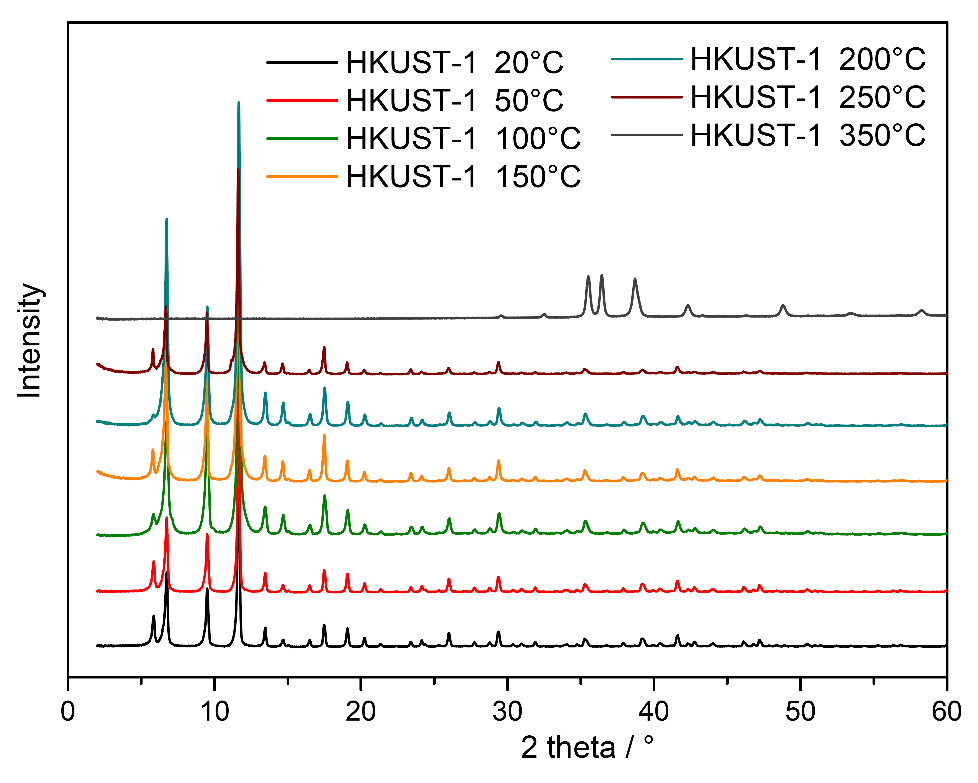


**Figure S3** Heating PXRD patterns of HKUST-1 measured at 20, 50, 100, 150, 200, 250 and 350 °C.


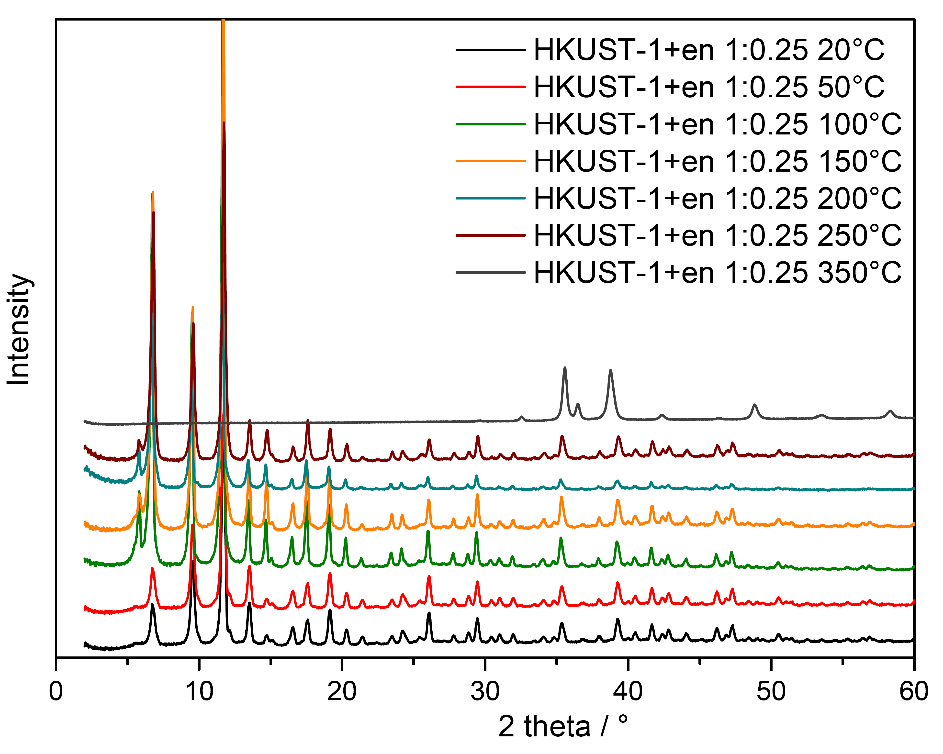


**Figure S4** Heating PXRD patterns of HKUST-1 : *en* / 1: 0.25 measured at 20, 50, 100, 150, 200, 250 and 350 °C.


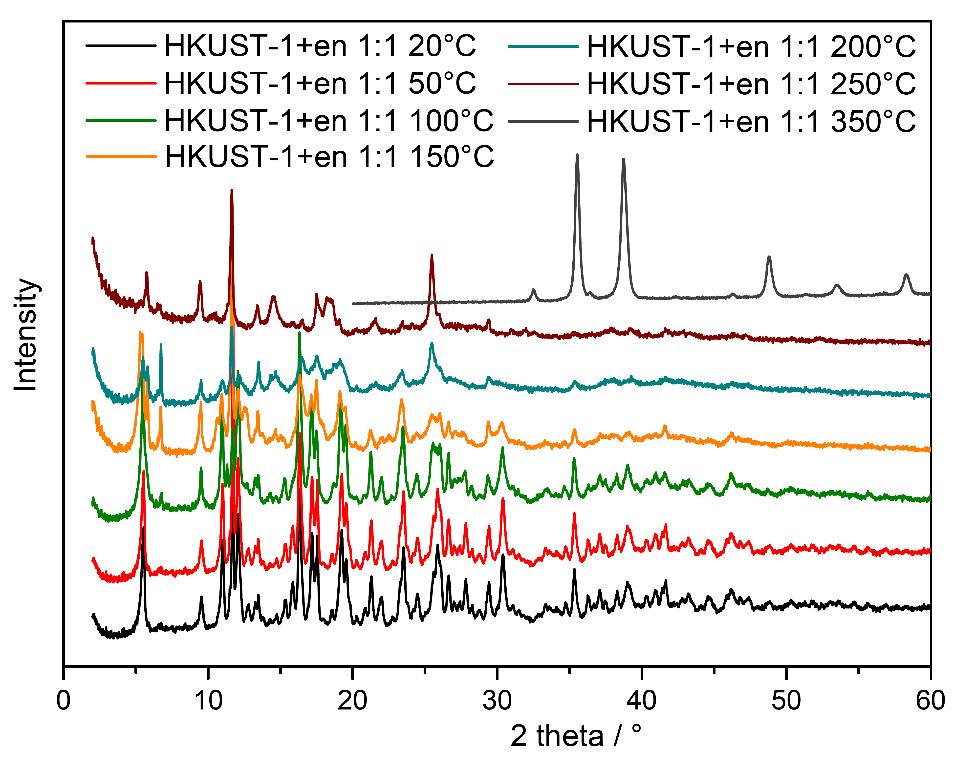


**Figure S5** Heating PXRD patterns of HKUST-1 : *en* / 1: 1 measured at 20, 50, 100, 150, 200, 250 and 350 °C.


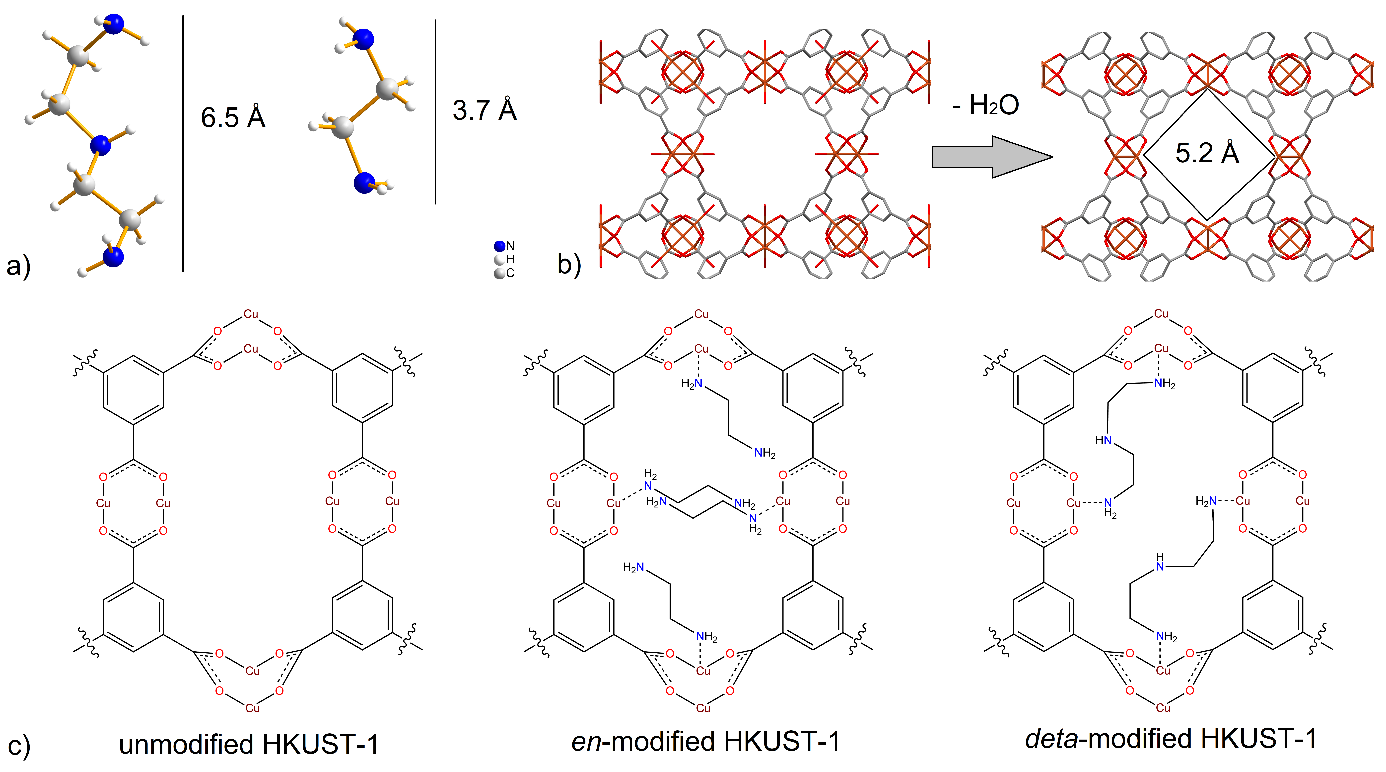


**Figure S6** Molecular size of *en* and *deta* amine. b) Formation of CUSs and the distance of CUSs within the HKUST-1 framework. c) Schematic of the entrance pore window in HKUST-1 and the coordination modes of *en* and *deta* molecules.


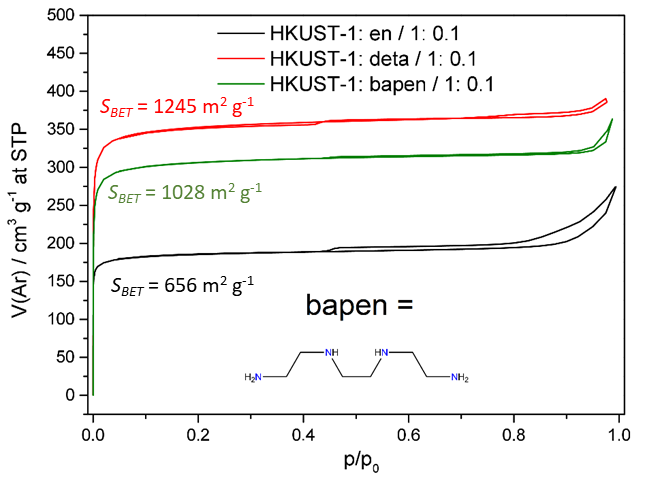


**Figure S7** Argon adsorption/desorption isotherms of amine-modified materials: HKUST-1: *en* / 1: 0.1 (black line), HKUST-1: *deta* / 1: 0.1(red line) and HKUST-1: *bapen* / 1: 0.1 (green line). The inset shows the molecular structure of *bapen* molecule.

**Tables:**

**Table S1** Assignment of characteristic absorption bands in the infrared spectra of prepared materials.

|  | ***ν*(NH)** | ***ν*(CH)_al_** | ***δ*(NH)** | ***ν*(COO^-^)_as_** | ***ν*(COO^-^)_s_** | ***δ*(CCH)_ar_** | ***δ*(COO^-^)** |
| --- | --- | --- | --- | --- | --- | --- | --- |
| HKUST-1 : *en* / 1: 0.1 | - | 2950 2840 | 1624 | 1561 | 1367 | 1110 | 727 |
| HKUST-1 : *en* / 1: 0.25 | 3271 3154 | 2981 2945 2892 | 1620 | 1564 | 1370 | 1112 | 725 |
| HKUST-1 : *en* / 1: 0.5 | 2368 3154 | 2980 2949 2896 | 1618 | 1562 | 1351 | 1103 | 728 |
| HKUST-1 : *en* / 1: 1 | 3246 3150 | 2972 2947 2924 2888 | 1616 | 1562 | 1339 | 1098 | 723 |
| HKUST-1 : *en* / 1: 1.5 | 3248 3148 | 2970 2947 2927 2884 | 1615 | 1563 | 1337 | 1102 | 723 |
| HKUST-1 : *deta* / 1: 0.05 | - | 2978 | 1643 | 1549 | 1367 | 1111 | 728 |
| HKUST-1 : *deta* / 1: 0.1 | - | 2976 2924 | 1643 | 1547 | 1368 | 1112 | 729 |
| HKUST-1 : *deta* / 1: 0.25 | 3241 3153 | 2977 2921 2877 | 1642 | 1546 | 1367 | 1094 | 728 |
| HKUST-1 : *deta* / 1: 0.5 | 3240 3155 | 2974 2923 2875 | 1632 | 1541 | 1355 | 1092 | 721 |
| HKUST-1 : *deta* / 1: 0.75 | 3241 3152 | 2972 2922 2875 | 1630 | 1542 | 1346 | 1092 | 718 |
| HKUST-1 : *deta* / 1: 1 | 3239 3153 | 2978 2921 2873 | 1631 | 1542 | 1345 | 1093 | 717 |

- – not observed, s – symetric, as – asymetric, al – aliphatic, ar – aromatic
